# Oral Health Knowledge and Behaviors among Adolescents with Type 1 Diabetes

**DOI:** 10.1155/2010/942124

**Published:** 2010-05-13

**Authors:** Valerie A. Orlando, Lonnie R. Johnson, Anne R. Wilson, David M. Maahs, R. Paul Wadwa, Franziska K. Bishop, Fran Dong, Elaine H. Morrato

**Affiliations:** ^1^Department of Community and Behavioral Health, Colorado School of Public Health, University of Colorado Denver, Aurora, CO 80045, USA; ^2^Department of Surgical Dentistry, School of Dental Medicine, University of Colorado Denver, Aurora, CO 80045, USA; ^3^Department of Pediatric Dentistry, School of Dental Medicine, University of Colorado Denver, Aurora, CO 80045, USA; ^4^Barbara Davis Center for Childhood Diabetes, University of Colorado Denver, Aurora, CO 80045, USA; ^5^Department of Pediatrics, School of Medicine, University of Colorado Denver, Aurora, CO 80045, USA; ^6^Children's Outcomes Research Program, The Children's Hospital, University of Colorado Denver, Aurora, CO 80045, USA; ^7^Department of Health Systems Management and Policy, Colorado School of Public Health, University of Colorado Denver, Mail Stop B119, Building 500, Third Floor, 13001 E. 19th Place, Room E3311, Aurora, CO 80045, USA

## Abstract

Early onset and more advanced periodontal disease has been reported for children with diabetes. We surveyed oral health knowledge, attitudes, and behaviors among adolescents with diabetes in order to inform potential intervention strategies. Study subjects were youth (ages 12–19 years) with type 1 diabetes (*N* = 90) participating in a cohort study investigating determinants of periodontal disease at a regional pediatric diabetes specialty clinic. Over 90% of the youth had been instructed on how to brush and floss and had preventive dental care in the past year. However, 44% knew that periodontal disease is associated with diabetes and 32% knew that it can start in childhood with bleeding gums. Despite being at high risk for developing periodontal disease, the mean toothbrushing frequency was once per day and 42% did not floss. Significant opportunity exists for improving periodontal disease knowledge and adoption of preventive oral hygiene behaviors in adolescents with diabetes.

## 1. Introduction

The World Health Organization estimates that more than 180 million people worldwide have diabetes mellitus, a number expected to double in the next twenty years [1]. In the United States, the prevalence of diabetes in children under 20 years of age is 2.0 cases per 1,000 [2], accounting for approximately 154,369 children in 2001 [3]. With its rising incidence, diabetes has captured the attention of specialist and generalist practitioners in both medicine and dentistry. Periodontal diseases have been termed “the sixth complication of diabetes mellitus” [4]. Researchers suspect that the link between diabetes and periodontal disease may be bidirectional; the body's response to periodontal pathogens may be exacerbated in individuals with diabetes *and* proinflammatory cytokines produced by gingival tissues during chronic periodontal infection may gain access to the bloodstream leading to increased insulin resistance and poor glycemic control [5, 6]. In a sample of adults with type 1 diabetes, periodontal disease severity was associated with both the duration of diabetes and the presence of diabetes complications [7]. The control of bacterial plaque through proper oral care is essential to overall systemic health and is becoming recognized as an important element in a comprehensive approach to treatment of the diabetic patient [8, 9]. 

Several reports have established the relationship between diabetes in children and periodontal disease [10–17]. Early investigations into the periodontal health of children with diabetes demonstrate expected correlations between poor metabolic control, as measured by percent glycated hemoglobin (HbA1c), and clinical dental plaque indices associated with gingival inflammation and bleeding [11, 18]. More recent studies indicate that periodontal disease and clinical attachment loss may begin during childhood for children with diabetes. Lalla and associates noted that the number of periodontally affected teeth in the youth aging 12–18 years and mean attachment loss was significantly higher in diabetic children compared with nondiabetic control subjects [14–16, 19]. In these studies, the average loss of clinical attachment among children with diabetes was comparable to the average attachment loss reported for United States adults ages 50–64 years [20]. Children with diabetes also had significantly more gingival inflammation than children in the control group even after adjusting for dental plaque as the primary etiology of gingivitis [15, 16]. 

Modifiable risk factors, such as oral health knowledge and oral hygiene behaviors, have not been as thoroughly investigated in adolescents with diabetes. Adherence to daily oral hygiene practices is important to prevent periodontal disease just as daily behaviors supporting glycemic control are crucial to minimize long-term risk for micro- and macrovascular complications in individuals with diabetes. Therefore, it is worthwhile to understand the level of oral health knowledge and behaviors among adolescents with diabetes in order to identify the most effective periodontal disease control and prevention strategies. A number of studies have investigated the relationship of certain psychological characteristics related to general oral health and diabetes care. These studies emphasized the underpinning social-psychological constructs critical to the development of bio-behavioral interventions [13, 21–26]. These investigations, however, did not examine behavioral and attitudinal factors specific to periodontal disease. 

The aim of our study was to survey periodontal health knowledge, attitudes, and behaviors among adolescents with diabetes in order to inform periodontal intervention strategies. The survey was modeled after standardized questions from US public health surveys to permit comparison with the general population. This study was part of a larger effort to develop interdisciplinary research focused on oral-systemic disease connections and the potential for improving patient care through such collaborations. 

## 2. Methods

### 2.1. Study Population

The Barbara Davis Center for Childhood Diabetes is a pediatric diabetes specialty clinic in Aurora, Colorado, colocated on the Anschutz Medical Campus with The Children's Hospital and the Schools of Medicine and Dentistry. The Barbara Davis Center Pediatric Clinic cares for approximately 3,000 children with diabetes from the Denver metropolitan area and the surrounding region and the patient population is considered representative of children with type 1 diabetes in the region. Our patient population is derived from an epidemiology cohort study investigating the prevalence and determinants of periodontal disease among adolescents aging 12–19 years with type 1 diabetes receiving their diabetes care at the Barbara Davis Center. Study subjects (*N* = 89) have been diagnosed with type 1 diabetes for at least 5 years. 

### 2.2. Oral Health Questionnaire

The forty item survey contained several questions modeled after U.S. national public health surveys including the National Survey of Children's Health (NSCH) 2003 [27] and 2007 [28], the Medical Expenditure Panel Survey (MEPS) [29], and the Centers for Disease Control Periodontal Surveillance Survey [30]. Items included self-reported oral health and dental conditions, history of dental treatment and preventive care, dental insurance coverage, and common dental home care behaviors. Knowledge of periodontal risk factors recognized by the American Academy of Periodontology [31] was also ascertained. We also asked participants to tell us about sources of health and dental care advice and their opinion of the relative importance of dental and periodontal care.

Items were written so as to be understood at a 6th grade reading level and the questionnaire was piloted with a small group of adolescents, taking each child on average less than 5 minutes to complete. The study received approval from the Colorado Multiple Institutional Review Board. After obtaining informed consent from participants and their parents, the Oral Health Questionnaire was administered along with other study questionnaires by the study coordinator. The oral health questionnaires were completed prior to the dental evaluation to minimize knowledge and/or response bias. 

### 2.3. Analytic Strategy

Descriptive statistics were performed to characterize sociodemographic and clinical characteristics of the study respondents, including age, sex, ethnicity, age at diabetes diagnosis, glycated hemoglobin levels (HbA1C), and Body Mass Index z-score (BMI z-score), a measure that reflects an individual's BMI relative to the general population and was calculated using a statistical algorithm from the United States Centers for Disease Control (http://www.cdc.gov/growthcharts/computer_programs.htm). 

The analysis plan included a comparison of several of the items to the corresponding norms found in National US Public Health Surveys that are referenced in the data tables below. For the NSCH- and MEPS-related questions, we report data from the subgroup analysis of ages 12–17 years. Chi-square tests were used for the responses with more than two levels. For dichotomous variables, confidence intervals were calculated using Proc Freq procedures with the binomial option using SAS software. Analyses were performed using SAS software (version 9.1; SAS Institute) [32].

## 3. Results

Table 1 presents characteristics of the survey respondents. On average, the adolescents had been diagnosed with type 1 diabetes for 8.4 years (standard deviation = 2.9 years) and their current HbA1C level was 8.9%. The adolescents were predominantly males (59.6%) and nonHispanic, whites (88.5%); the mean age was 14.9 years (standard deviation = 1.9). The vast majority of participants reported having a source of dental insurance to help pay for routine dental care (86.5%). 

Table 2 summarizes the adolescents' self-reported perceptions of their general oral health and the prevalence of specific dental problems. Nearly half of the adolescents rated the health of their teeth as being excellent or very good (48.3%), over 39.3% ranked the health of their teeth as good, and 12.4% as fair or poor. These rankings are significantly lower than the national norms reported in the 2007 National Survey of Children's Health, in which 70.6% of parents/caregivers rated the health of their adolescent's teeth as excellent or very good. We also asked participants to rate the health of their gums, and the ratings were similar to ratings they assigned to the health of their teeth. 

When asked about specific problems with teeth and gums, the greatest proportion of respondents indicated having crooked teeth or orthodontic concerns (41.5%), while the smallest proportion of respondents indicated having pain or toothache (5.8%). Thirty-three percent of adolescents with type 1 diabetes stated that plaque or tartar build up was a problem, while only 3.6% reported this in the nationally representative survey sample of the general population [27]. About thirty-two percent of adolescents with type1 diabetes reported bleeding gums, which was significantly higher than the national average of 4%. Cosmetic concerns, including discoloration or staining of teeth, also rated significantly higher among study subjects at 40.7% versus only 3.6% nationally. 

The frequency and type of dental care reported is shown in Table 3. In this sample of adolescents with type 1 diabetes, 93.2% reported having had a preventive dental visit within the past 6 months; and nearly all had seen a dentist within the last year. A higher percentage of children in the study reported having seen a dentist in the last 6 months than the national average; although this did not achieve statistical significance (*P* = .12). Approximately 81.4% of these adolescents had caries experience and had subsequently received restorative dental care. 

**Figure 1:**
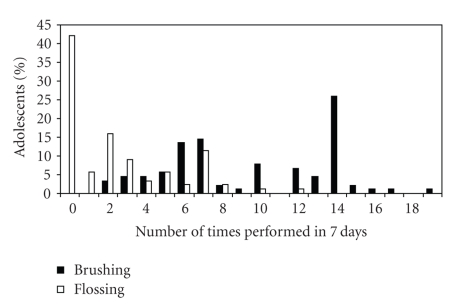
Frequency of toothbrushing and flossing.

With regard to individual oral hygiene behavior, the frequency of toothbrushing and dental flossing showed wide variability in this group of adolescents as illustrated in Figure 1. Most respondents appear to have regular daily toothbrushing habits, while fewer than half have adopted daily flossing. The median frequency for toothbrushing was 10 times in 7 days (interquartile range = 6–14). The median frequency for flossing was 2 times in 7 days (interquartile range = 0–4). 

As shown in Table 4, adolescents in the sample reported an overall greater exposure to advice and information from health care providers than did individuals in the national sample from the Medical Expenditure Panel Survey [29]. Advice about physical activity, wearing seat belts when driving or riding in a car, and wearing a helmet when riding a bicycle or motorcycle was reported by adolescents in our study at a rate nearly double that of the general population. Table 4 also reports health advice received from a dental professional. Seventy-seven percent of those participating indicated that doctors or health care providers had advised them about having regular dental checkups, as compared to only 40% of adolescents in the general population receiving this message. Among those surveyed, 92.0% reported receiving instructions about toothbrushing technique and 94.3% reported having been instructed about the use of dental floss. 

Fewer study participants reported specific knowledge about the etiology and factors contributing to gum disease (Table 4). Only 22.1% reported knowing that gum disease was infectious, and just 27.5% were aware that gum disease may begin in childhood with red and bleeding gums. A slightly greater percentage (44.0%) reported knowing that gum disease was more common in people with diabetes. Messages about the hazards of tobacco use and its effects on the oral cavity reached 69.8% of this group.

The relative importance of gum and teeth health when compared to medical health is shown in Table 5. When asked about the importance of taking care of their teeth, 79.8% of adolescents in our study strongly agreed with the statement “Taking care of my teeth is important”, while a smaller percentage (67.4%) strongly agreed that taking care of their gums was important. However, only half of the participants stated that taking care of their teeth and gums was as important as taking care of their medical health. 

## 4. Discussion

In a sample of adolescents with type 1 diabetes being treated at a pediatric diabetes specialty clinic, we found high utilization of professional preventive dental care services, and over 90% of these adolescents had received preventive dental care instructions from a dental professional on how to floss and brush their teeth. In contrast, though, there was low knowledge about risk factors for periodontal disease. Most notably, less than half of the adolescents were aware that periodontal disease is associated with diabetes and only one-quarter knew that periodontal disease can start in childhood with bleeding gums. Despite being a group of youth at high risk for developing periodontal disease, the average toothbrushing frequency was just 9.5 times per week and nearly 42% of these adolescents did not floss. These adolescents reported that taking care of their gums was less important than taking care of their teeth and both had lower priority than taking care of their medical health. If confirmed, our findings suggest several opportunities for improving clinical dental practice in the area of health education, intervention planning, and medical-dental professional collaboration to improve the oral health outcomes for young people living with type 1 diabetes. 

First, with regard to raising periodontal disease awareness and knowledge, it would appear that adolescents with type 1 diabetes are an audience of informed young people who are aware of general health and prevention concepts. For example, messages about healthy eating and physical activity were reported by a significantly higher percentage of study group adolescents when compared to national norms for preventive health advice reported by the Medical Expenditure Panel Survey [29]. Knowledge about the oral health risks of smoking also seems high in this group of adolescents with type 1 diabetes. While we might expect more vigilance in patient education on these topics from diabetes care providers and educators, it was interesting that a greater proportion of adolescents in our study group reported significantly more preventive health advice in other topics as well. Our findings suggest, however, that most youth with diabetes may not be aware of their increased risk for periodontal disease. This is similar to the findings of Moore and colleagues, noting that many adult diabetic patients “lack(ed) important knowledge about the oral health complications of their disease” [34]. Therefore, dental professionals should work with diabetes educators and incorporate periodontal-specific oral health messages into routine diabetes education for adolescents.

A second opportunity is to actively promote optimal oral hygiene habits for disease prevention and control through effective toothbrushing and flossing. Despite being instructed by a health professional on how to floss, the majority of the adolescents engaged in little or no flossing behavior. Youth who are self-responsible for their diabetes care should be encouraged to adopt increased dental flossing to control gingivitis and to establish healthy habits to prevent periodontitis. Relatively simple interventions may be effective, particularly when proven behavioral change techniques are used, such as supporting the development of personal behavioral intentions as described by McCaul et al., and more recently by Sniehotta et al. [26, 35, 36]. Research by Syrjälä et al. has also explored the construct of self-efficacy and the theory of reasoned action in describing determinants of oral health and diabetes self-care behaviors for adults with type 1 diabetes [23, 25, 26]. Self-efficacy is a person's belief in his or her ability to succeed in a particular situation. Individuals with diabetes who were confident in their ability to manage their diabetes were also more likely to adhere to oral hygiene recommendations [25]. Though nearly all participants reported high rates of dental attendance and receiving toothbrushing and flossing instructions from dental professionals, perhaps the motivation to practice these health behaviors, particularly flossing, has not been as clearly connected with the outcomes for gingival health or the consequences of periodontal disease for diabetic patients. Promoting dental attitudes and subjective norms among diabetic children may improve the likelihood of practicing effective oral hygiene behaviors such as flossing [26]. 

Not surprisingly, the young people who participated in our study appear to be concerned with cosmetic factors and the appearance of their teeth. Self-esteem and peer acceptance as well as family environment have been shown to influence oral hygiene behaviors with adolescents [37, 38]. Further study of social and psychosocial factors could provide insight into ways to motivate teens to improve brushing and flossing behaviors. 

When considering the rating of dental health, it is interesting to note that this particular group of adolescents with diabetes did not rate their oral health as favorably as the general population. Perhaps children with type 1 diabetes take a more critical view of their oral health status or are less likely to rate any aspect of their health as excellent in light of their systemic health problem. However, an older survey collected for the Third National Health and Nutrition Examination Survey found that half of adolescents reported excellent or very good dental health, which is similar to our observations [39]. Interim analysis of the clinical periodontal findings within this group is presently underway and should provide a more comprehensive picture of the oral health status and how perception compares with clinical measures. 

This study has limitations. Results from a single diabetes specialty clinic may not reflect the health knowledge, attitudes, and behaviors of a typical adolescent with diabetes, although the children seen at the Barbara Davis Center are representative of children with type 1 diabetes in the region. However, adolescents in our study were more likely to have had preventive dental care in the past year than an average adolescent in the U.S. More research is needed to confirm our findings; however, there are some clinical indicators that suggest that our sample may be representative of general health behaviors in youth with diabetes. For example, our population's level of glycemic control was comparable to rates observed across large international samples of diabetic youth [40, 41]. The BMI scores of our population were also similar to the norms for children of this age [41], and the self-reported rates of caries were also comparable to rates reported from the National Survey of Children's Health for this age group [27]. 

Self-reported data also limit our ability to validate these findings. Response bias may have influenced individuals to overreport dental visit attendance and brushing or flossing behaviors. The national normative data are provided to offer context for our results. Caution should be applied when making direct comparisons between our findings and national normative data. For example, the National Survey of Children's Health obtains data via telephone interview surveys and asks parents to describe the health and dental circumstances of their children [27, 28]. It is likely that children and adolescents report their perceptions of oral health and the need for dental treatment based on oral signs and symptoms, and that assessment may differ from the perceptions and reports of their parents [42]. 

## 5. Conclusion

Though they have significantly greater risk for the development of periodontal disease and despite having received regular professional dental care and instructions, the adolescents with type 1 diabetes in our study reported suboptimal oral hygiene behaviors. It is also important to note that this population of patients has a lower opinion of their self-reported oral health status and may require more vigilant instructions and motivation to perform preventive behaviors. 

Our findings suggest that a significant need exists for improving periodontal disease knowledge and adoption of preventive oral hygiene behaviors that result in improved oral health for adolescents with diabetes. As dental practitioners, we must recognize the opportunity to contribute in a meaningful way to health promotion for children with diabetes through early disease detection, vigilant dental maintenance, monitoring of blood glucose levels, nutritional counseling, and ongoing collaboration with medical providers for the optimal health of the patient. 

## Figures and Tables

**Table 1:** Select characteristics of participants.

	Adolescents with type 1 diabetes (*N* = 89)	

Demographic characteristics	%	(*n*)
Sex		
Male	59.6	(53)
Female	40.4	(36)
Race		
White/Caucasian	95.4	(83)
Other	4.6	(4)
Ethnic origin		
Hispanic	11.5	(9)
Non-Hispanic	88.5	(69)
Dental insurance status		
Yes	86.5	(77)
No	7.9	(7)
Do not know	5.6	(5)

Clinical characteristics	Mean	(SD)

Age	14.9	(1.9)
No. of years since diabetes diagnosis	8.4	(2.9)
Body mass index z score (BMIz)	0.53	(0.8)
Glycated hemoglobin (HbA1C)	8.9	(1.5)

**Table 2:** Self-perception of oral health.

	Adolescents with type 1 diabetes		U.S. National Norm	
	%	*n*	%	*P* *-*value
How would you rate the health of your teeth?*				
Excellent/very good	48.3	43	70.6	
Good	39.3	35	21.5	<.001
Fair/poor	12.4	11	7.9	
How would you rate the health of your gums?			NA	
Excellent	10.2	9		
Very good	33.0	29		
Good	43.2	38		
Fair	12.5	11		
Poor	1.1	1		

	%	95% CI	%	*P*-value

What specific problems, if any, do you have with your teeth and gums?				
Pain/toothache*	5.8	1.9–12.9	10	.19
Cavities/decayed teeth or cavities*	25.9	16.8–36.9	19.4	.14
Bleeding gums*	31.8	22.3–42.6	4.0	<.001
Cosmetic concerns: discoloration**	40.7	30.2–51.8	3.6	<.001
Orthodontic concerns: crooked teeth**	41.5	30.7–52.9	33.5	.13
Bad breath	26.7	17.8–37.4	NA	
Plaque or tartar buildup on teeth**	33.3	22.9–45.2	3.6	<.001

NA indicates that national normative data were not available.

*U.S. comparison data from the 2007 National Survey of Children's Health [28]. The survey asked about specific problems within the past 6 months.

**U.S. comparison data from the 2003 National Survey of Children's Health [27]. The survey asked about specific problems if parents reported that teeth were in fair or poor condition. Bleeding gums was phrased as “gum problems”.

**Table 3:** Professional dental care.

	Adolescents with type 1 diabete		U.S. National Norm	
	%	95% CI	%	*P*-value
Professional dental care visits				
Preventive care in the past 12 months	93.2	85.8–97.5	87.8	.12
(check-ups, screenings, and sealants)*				
Restorative care (cavities, fillings)	81.4	71.6–89.0	NA	
Periodontal treatment (scaling and	2.3	0.3–8.2	NA	
root planning, or “deep” cleaning)				
Orthodontic treatment	47.7	37.0–58.6	NA	
Cosmetic (whitened teeth)	19.5	11.6–29.7	NA	

	%	*n*	%	*P*-value

How long has it been since you last saw a dentist?*				
≤6 months	81.8	72	70.7	
6 months–1 year	13.6	12	15.6	.06
1-2 years	3.4	3	7.7	
>2 years	1.1	1	6.0	

NA indicates that national normative data were not available.

*U.S. comparison data from the 2007 National Survey of Children's Health [28].

**Table 4:** Sources of health information and advice.

	Adolescents with type 1 diabetes (*N* = 89)		U.S. National Norm*	
	%	95% CI	%	*P-*value
Have your doctors or other health care providers ever discussed or given you advice about the following?				
Having regular dental check-ups	77.1	66.6–85.6	40.3	<.001
Eating healthy	87.6	79.0–93.7	48.9	<.001
Physical activity	79.3	69.3–87.3	40.6	<.001
Wearing seat belts when driving/riding in a car	64.7	53.6–74.8	30.9	<.001
Wearing a helmet when riding a bicycle/motorcycle	66.7	55.8–76.4	31.3	<.001

Has a dentist, dental hygienist, or another dental professional ever			NA	
Given you instructions for how to brush your teeth	92.0	84.1–96.7		
Given you instructions for how to floss	94.3	87.2–98.1		
Told you that you have gum problems, gum	22.1	13.9–32.3		
infections, or gum inflammation				
Told you that you lost bone around your teeth?	1.1	0.0–6.2		

Did you discuss at school or has a doctor, dentist, or another health care professional ever told you that gum disease			NA	
Is more common in people with diabetes	44.0	33.2–55.3		
Can start in childhood with red and bleeding gums	27.5	18.1–38.6		
Is an infection caused by germs that can be passed	23.2	14.6–33.8		
from person to person in your saliva				
Can be caused by using tobacco	69.8	58.9–79.2		
Is related to how much stress you have	14.6	7.8–24.2		
Can be caused by grinding your teeth at night?	47.1	36.1–58.2		

NA indicates that national normative data were not available.

*U.S. comparison data from the Medical Expenditure Panel Survey, 2000–2004 [33].

**Table 5:** Importance of oral health for adolescents with type 1 diabetes.

	Percent agreeing with statement (*N* = 89)				
	Strongly agree	Somewhat agree	Somewhat disagree	Strongly disagree	Do not know
Taking care of *my teeth* is important	79.8	20.2	0	0	0
Taking care of *my teeth* is as important as taking care of my medical health	51.1	43.2	3.4	0	2.3
Taking care of *my gums* is important	67.4	31.5	0	0	1.1
Taking care of *my gums* is as important as taking care of my medical health	47.2	43.8	5.6	0	3.4
